# Internal fixation of fractures of the shaft of the humerus by dynamic compression plate or intramedullary nail: A prospective study

**DOI:** 10.4103/0019-5413.33685

**Published:** 2007

**Authors:** S Raghavendra, Haresh P Bhalodiya

**Affiliations:** Department of Orthopedics, B. J. Medical College and Civil Hospital, Ahmedabad, India

**Keywords:** Fractures of the shaft of the humerus, intramedullary nailing, plating

## Abstract

**Background:**

The indications for surgical management of fractures of the shaft of the humerus are clear, but selecting the right implant for internal fixation of humeral fractures has been a dilemma.

**Materials and Methods:**

Thirty-six patients (mean age 40.53 years) with fractures of the shaft of the humerus were followed for 12 to 24 months in a prospective study. Eighteen patients each underwent open reduction and internal fixation with compression plating and ante grade interlock nailing. Clinical and radiographic outcome measures included fracture healing, shoulder and elbow functions, need for additional procedures and any complication such as infection and recovery of radial nerve palsy. The results were analyzed statistically using the SPSS 11.5 software, with parametric and nonparametric tests.

**Results:**

Nine of the fractures treated with compression plating and seven of those treated with interlock nailing achieved union within six months. Though there was no significant difference in union time between the treatment groups, patients operated with interlock nailing underwent more number of secondary bone grafting procedures to obtain union (six against two). There were 12 patients (66.6%) with excellent and good results in the plating group compared to four patients (25%) in the nailing group. Interlock nailing was associated with significant reduction in shoulder function (*P*=0.03) and in overall results (*P*=0.02).

**Conclusion:**

Though there was no significant difference between plating or nailing in terms of time to union, compression plating is the preferred method in the majority of fractures of the shaft of the humerus with better preservation of joint function and lesser need for secondary bone grafting for union.

Most of the fractures of the shaft of the humerus are best treated nonoperatively.^1^–^12^ Numerous authors^1^–^12^ have highlighted the advantages of conservative, gravity-dependent treatment of these fractures by bracing in ambulatory patients^13^ preceded by short period of traction.^13^ Operative fracture stabilization carries risk of infection and iatrogenic radial nerve injury.^5^,^9^

Despite this, operative stabilization is warranted in Multiple-injury patients,^1^,^5^,^10^,^13^,^15^–^19^ segmental humeral fractures, fractures with concomitant ipsilateral forearm fractures, a so-called “floating elbow”^15^,^19^,^20^ and inability to maintain fracture alignment with nonoperative treatment (either due to angulation or noncompliance in obese or elderly patients).^2^,^5^,^6^,^9^,^14^,^18^,^21^,^22^ Fixation of a fracture of the humeral shaft in the Multiple-injury patient is said to increase the mobility of the patient, simplify the difficult nursing care in the intensive care unit and permit full access to the patient for pulmonary physiotherapy.^10^ Fixation also controls the angulation and length of the fracture in a supine, unconscious patient and allows full, early mobilization of the upper extremity.^10^,^15^,^23^

Selecting the right implant for internal fixation of humeral fractures remains controversial. Presently, to the best of our knowledge, there are only four published studies in the English language literature with limited number of patients which compare operative results between plating and interlock nailing^24^–^27^ with contradictory conclusions. We hereby present a prospective comparative study of humerus fracture internally fixed with dynamic compression plate or intramedullary nail.

## MATERIALS AND METHODS

Thirty-six consecutive patients operated with either compression plating or interlock nailing for acute fractures of shaft humerus during the period of 2000 to 2003 with minimum follow-up of 12 months were included in the present analysis. Eighteen patients each underwent open reduction and internal fixation with compression plating, and ante grade interlock nailing (with open reduction in three patients). All acute diaphyseal fractures included in our study were either closed or open Gustilo Grade I.^28^ Patients with fractures of the proximal and distal humerus (extraarticular fractures in the proximal and distal 5cm of the bone) and pathological fractures, were not included in our study. Clinical details are presented in Table 1.

**Table 1:** Clinical details of patients and fracture characteristics

Number	Plating group 18	Nailing group 18	Total 36
Sex			
Male	17	15	32
Female	1	3	4
Age (years)			
Mean	40.83	40.22	40.53
Range	22.70	18-70	18-70
Mechanism of injury			
Road traffic accident	7	9	16
Fall	9	7	16
Assault	2	2	4
Fracture type			
Open grade I	3	2	5
Closed	15	16	31
Fracture position			
Lower 1/3	3	5	8
Middle-lower 1/3	2	2	4
Middle 1/3	12	8	20
Middle-upper 1/3	0	2	2
Upper 1/3	1	1	2
AO subtype			
A1	2	0	2
A2	7	8	15
A3	7	3	10
B2	2	7	9
Preoperative radial nerve injury	3	1	4
Associated injury	4	4	8

The surgeries were performed between six hours to three weeks after the initial injury. Primary bone grafting (n=8) was done when bone loss or comminution was present.^19^

During compression plating, we used the posterior approach in 15 patients, with the patient in the lateral position and an antero-lateral approach in a supine position was used in three patients. The choice of the approach was based on fracture position and morphology. A 4.5 mm compression plate (DCP (n = 11) and limited contact DCP (n = 7)) was used in all patients. Interfragmentary compression by means of lag screws was used when required. Generally, a plate that permitted screw fixation to at least six cortices both in the proximal and in the distal fragment was used.^19^,^29^

**Figure 1:**
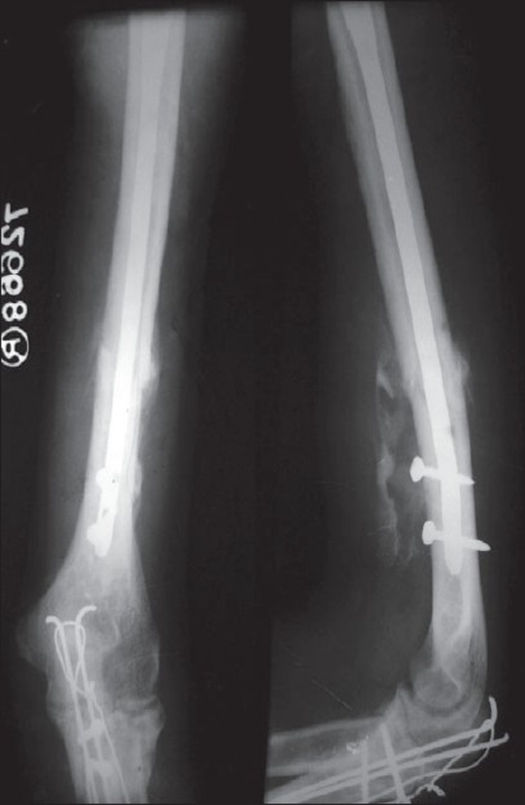
Follow up X-ray of a right arm in 9 patient in nailing group shows union of right humerus with ipsilateral olecranon fracture. The union occured in 14 weeks

The ante grade interlock nailing [Figure 1] was used in the study. There are reports to suggest interlock nailing for fractures in any part of the humerus,^18^ but we have used this technique for fractures of the middle 60% of the shaft.^17^

Postoperatively all patients were initiated on active shoulder and elbow mobilization exercises. Periodic radiographic evaluation was carried out to look for union,^14^,^19^ to assess the need for additional procedures and to check for complications. All patients were evaluated on the basis of the outcome criteria [Table 2]. When any two different criteria fell into separate categories, the lower category was selected to classify the outcome.

**Table 2:** Criteria for evaluation

Criterion	Excellent	Good	Fair	Poor
Union	Uneventful	Uneventful	Secondary procedure	Nonunion
Radial nerve palsy	Nil	Transient	No recovery	Disabling, need foraddl. procedure
Infection	Nil	Superficial	Deep	Osteomyelitis
Joint movement				
Shoulder	Normal	>75%	50-75%	<50%
Elbow	Normal	>75%	50-75%	<50%
Occupation	Same	Same	Changed	Left

### Statistical analysis:

The results were analyzed statistically using the SPSS 11.5 software with student's t test and nonparametric tests (Fisher's exact). The value of alpha was set at 0.05.

## RESULTS

We have evaluated our patients based on fracture healing, functional restoration of the limb and presence of complications and need for additional procedures. Two patients in the nailing group were lost to follow-up.

All patients in our study achieved union. The majority of our patients (all the 18 patients in the plating group and 13 in the nailing group) achieved union within one year of initiation of treatment; three fractures treated with nailing achieved union after one year. Nine of the fractures treated with compression plating and seven of those treated with interlock nailing achieved union within six months (*P*=0.744, Fisher's exact test). The average time to union was 25.9 weeks (SD=7.28) in the plating group and 34.6 weeks (SD=20.34) in the nailing group (*P*=0.12), but the patients operated with interlock nailing underwent more number of secondary bone grafting procedures (six versus two) to obtain union though this difference was not statistically significant. (*P*=0.10; Fisher's exact test).

One patient treated with compression plating had an implant failure three months later and underwent implant removal, refixation with interlock nailing and secondary bone grafting and had subsequent union. One patient, who was eventually lost to follow up after 1 month, operated with interlock nailing for a lower third fracture of the shaft had iatrogenic communition of the distal fragment during nailing and required open reduction, encerclage wiring and primary bone grafting and had superficial infection postoperatively. Impingement of the nail at the acromion was noted in two patients and one of them underwent reinsertion of nail.

The range of motion of both the shoulder and elbow joints were compared from the opposite side [Table 3]. In comparison to plating, patients operated with interlock nailing had significant restriction of shoulder movement (*P*=0.03; Fisher's exact test), the difference in the restriction of the elbow range of motion was not significant among the groups (*P*=0.72).

**Table 3:** Range of motion of shoulder and elbow

	Shoulder					Elbow			
		
	Normal	<75%	50-75%	<50%	Normal	>75%	50-75%	<50%
Nailing	8	2	4	2	12	3	1	0
Plating	16	1	0	1	14	3	1	0

**Figures 2 and 3:**
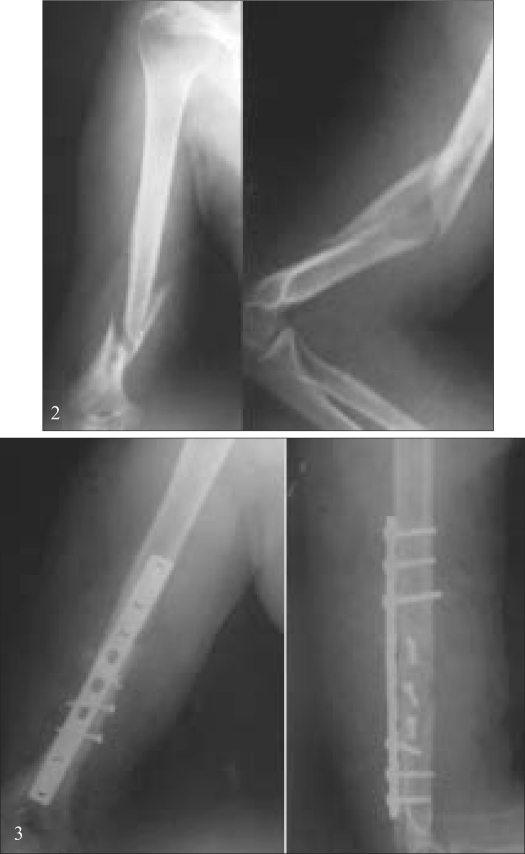
Pre and post operative radiographs of a patient (plating group) who underwent plating for a 3 week old comminuted fracture of right humerus. The patient had post-operative neuropraxia of the radial nerve which recovered completely on conservative treatment. The patient had union at 21 weeks

Radial nerve palsy [Figures 2 and 3] was present in four patients after injury (11.1% incidence). Of the three patients who had undergone ORIF with compression plating with associated nerve injury, one had full recovery of function and one had partial but useless recovery of motor function and the third didn't recover and did not come for treatment after union of fracture. All these patients were found to have an intact nerve during peroperative exploration. One patient treated with interlock nailing had no recovery of preoperative radial nerve palsy and underwent tendon transfer to improve function; the nerve was found buried in fibrous callus when it was explored at a later date. There were no cases of postoperative radial nerve palsy after interlock nailing, while one patient who had a neuropraxia after plating had full recovery on conservative treatment.

The overall results according to the outcome score are given in Table 4. For statistical analysis, we have grouped results considered excellent and good (E+G) and compared them with those considered fair and poor (F+P). Patients operated with plating fared significantly better than those operated with interlock nailing when the overall results were analyzed (*P*=0.02; Fisher's exact test).

**Table 4:** Overall results in various groups

Result	Plating	Nailing
Excellent (E)	8	1
Good (G)	4	3
Fair (F)	5	9
Poor (P)	1	3

## DISCUSSION

The indications for surgical management and internal fixation of fractures of the shaft of the humerus are clear.^1^,^2^,^5^,^6^,^9^,^10^,^12^–^16^,^18^,^20^–^22^,^30^ Compression plating has been regarded as the gold standard for operative treatment^31^ with high rates of fracture healing and consolidation^16^,^19^,^25^,^32^ and good outcome^16^ with no adverse effect of immediate full weight-bearing on fracture union or alignment.^32^

Advocates of intramedullary fixation have highlighted various disadvantages of open reduction and internal fixation with compression plating which requires extensive open surgery with stripping of soft tissues from bone,^18^ a longer operative time^5^ and less secure fixation, especially in the elderly with osteoporotic bone and if crutch walking is required.^5^,^18^ Hall *et al.,*^9^ have highlighted three complications associated with plating, namely infection, nonunion and radial nerve injury.^5^,^10^ Intramedullary fixation is reported to involve a simpler technique with minimal exposure^8^,^33^,^34^ and shorter operative time with less blood loss.^5^,^10^,^33^,^35^,^7^,^10^,^21^,^36^ The preservation of fracture hematoma, soft tissue and periosteum around the fracture that occurs with closed unreamed nailing has been proposed for high rates of union and good results,^4^,^9^,^10^,^33^ with no risk of iatrogenic radial nerve palsy.^31^,^37^ Locked nailing is said to provide a rotationally stable fixation and avoid the tendency of various unlocked nails to back out.^18^,^33^

Various authors have reported complications associated with intramedullary nailing of the shaft of the humerus. The anatomical configuration of the shaft of the humerus makes it prone for residual fracture site distraction,^7^,^10^,^21^,^36^ especially where the sagittal diameter of the distal part is small.^5^,^10^,^14^ Residual fracture site distraction can lead to increased risk of delayed union /nonunion,^9^,^10^,^14^,^21^,^36^,^37^ with the need for additional procedures to obtain union. Unlike in more tubular bones like the femur and tibia, interlock nailing has not been recommended as standard method of management for a humeral diaphyseal fracture.^5^,^24^,^25^ The findings in our study have also demonstrated the same.

Impairment of shoulder function^4^,^5^,^7^–^10^,^14^,^17^,^18^,^24^–^26^,^34^–^36^ as a consequence of ante grade intramedullary fixation has been attributed to various reasons. Proximal migration of unlocked or dynamically locked nails with impingement at the acromion^4^,^5^,^8^–^10^,^14^,^17^,^24^,^25^,^35^ and consequent impairment of abduction^8^,^9^ and external rotation^9^ is said to require a secondary procedure for the protruding devices,^5^,^9^,^10^,^14^,^17^ after which the range of motion increases.^14^ Moreover, ante grade nailing has been found to violate the rotator cuff,^5^,^7^,^9^,^10^,^14^,^24^ which has been confirmed by sonography of the cuff.^7^ A medial starting point is said to avoid the avascular area of the cuff and give a straight access to the medullary canal, without compromising the rotator cuff healing.^38^ Adhesive capsulitis of the shoulder has also been reported after ante grade nailing.^5^,^10^,^14^

There have been reports of impairment of elbow function after retrograde nailing,^8^–^10^,^14^ possibly due to myositis ossificans.^8^ There is an opinion that retrograde nailing can lead to iatrogenic distal end fracture, especially when attempted in fractures of the distal shaft.^10^,^18^,^21^

The results of this study demonstrate that though there was no statistically significant difference in the time required for union, patients operated with interlock nail underwent more number of secondary bone grafting procedures than those operated with compression plating. Interlock nailing was associated with significant restriction in shoulder movement (*P*=0.03) and a reduction in overall results (*P*=0.02). These findings are comparable to other prospective studies.^24^–^27^ The overall results were in favor of nailing in a study by Lin^24^ and in favor of plating in the study by McCormack.^25^

We are aware of the fact that we have recruited a fewer numbers of patients, which reduces the power (1-β) of the study. A larger randomized trial or may be a multi-center trial can further improve the interpretation of the results.
